# Academic hospitals in the Toronto region collaborate to optimize occupational health and safety

**DOI:** 10.1177/08404704241252910

**Published:** 2024-06-03

**Authors:** Anum Aftab, Tamara Dus, Christopher Aiken, Arlene Gladstone, Wendy Morgan, Nicholas Tomiczek, Laura Alexander

**Affiliations:** 1 7989 University Health Network, Toronto, Ontario, Canada.; 2 University of Toronto, Toronto, Ontario, Canada.; 3 Women’s College Hospital, Toronto, Ontario, Canada.; 4 Mount Sinai Hospital, Toronto, Ontario, Canada.; 5 Sunnybrook Health Sciences Centre, Toronto, Ontario, Canada.; 6 The Hospital for Sick Children, Toronto, Ontario, Canada.

## Abstract

In March 2020, as the COVID-19 cases began to rise in Ontario, Canada, the central role of Occupational Health and Safety (OHS) to ensure the well-being of hospital workforce became highly visible. While Ontario’s hospitals concentrated efforts to meet each challenging and uncertain wave stressing the system, it was apparent that there is a lack of consistency in best practices and policy response across the healthcare sector. Additionally, the unprecedented pressure on healthcare workforce as they attempted to meet the pandemic’s new surging demands resulted in workforce shortages and increased levels of burnout, making it difficult to engage, support, and retain the staff necessary for delivering highest quality of services. The Toronto Academic Health Science Network (TAHSN), a dynamic consortium of 14 healthcare organizations, established a collaborative to implement an integrated effort and align on structure, processes, and standards that will increase strength and defensibility of TAHSN programs. To foster community building, identify areas of common concern, and co-create practices during and beyond the COVID-19 pandemic, a structured network of 14 OHS directors across the healthcare organizations was established. This article discusses the origin of the TAHSN collaborative, the thriving community vision for partnership, and the case study methodology used to combine capabilities to showcase innovation and excellence in care together.

## Introduction

The World Health Organization (WHO) defines Occupational Health and Safety (OHS) as an area of work in public health that promotes and maintains highest degree of physical, mental, and social well-being of workers in all occupations. OHS in healthcare settings aims to support a best-in-class safe and healthy work environment for all Healthcare Workers (HCWs), including staff, physicians, learners, and volunteers. For many years, hospital OHS has been focusing on “traditional services” including (but not limited to) pre-employment health assessment, immunization, disability management, minor first aid for workplace injuries, and communicable disease surveillance programs. However, during the COVID-19 pandemic, it became evident that OHS was required to recognize and respond quickly to an emerging infection that was putting the lives of HCWs at an exponentially high risk and overwhelming the healthcare system like never before. In addition to the traditional services, OHS in hospitals was required to educate and support the HCWs with appropriate Personal Protective Equipment (PPE), conduct COVID-19 testing, complete contact tracing, and monitor staff affected by the virus until they were safe to return to work.

When the COVID-19 pandemic emerged, the central role of OHS to ensure the well-being of HCWs became highly visible. With each wave testing the resilience of the hospital sector and maximizing the finite capacity and resources, the pandemic challenged the existing health systems and health human resources, compromising timely access to care and peaked rates of sick time, overtime, and use of agency staff. As organizations attempted to meet these challenges, it was apparent that there is a lack of consistency in OHS practices across the healthcare organizations in Toronto, one of the most dynamic and richly diverse regions in the world. To cope with the pandemic and protect workforce welfare, each organization developed unique practices and response strategies resulting in the quality and consistency of service delivery varying dramatically between the 14 TAHSN organizations. This gap was specifically exacerbated due to inconsistencies in resources and systems that were predominantly shaped by the hospital size and subject to different funding. To urgently address the growing demand for OHS services in the face of the ongoing pandemic, TAHSN organizations partnered to align on practices and programs and enhance the existing service delivery model; foster collective excellence, innovation, and impact; and alleviate the additional pressure that had been placed on hospitals and the HCWs. Developing a competent collaborative intended to provide the healthcare organizations with an opportunity to establish a shared vision of centralizing decision-making to optimize, advance, and sustain the function of OHS, while also improving financial performance through shared programs: cost savings, revenues, and profits.

This article discusses the novel partnership between the TAHSN healthcare organizations to conduct a detailed assessment of current state practices within OHS that results in improved problem solving, peer learning, shared purpose, and advanced solutions. While leveraging internal data, the healthcare organizations aimed to discover unique strengths and challenges in order to develop a set of common strategic priorities in an overarching long-term improvement plan^1^ that would address the challenges of strained systems and sub-optimal staffing levels while closing gaps in the continuum of care.

### Toronto Academic Health Science Network

Toronto Academic Health Science Network is a dynamic consortium of the University of Toronto and its 14 affiliated academic health organizations providing leading edge research, teaching, and clinical care. As a leader in Canadian healthcare and one of the largest, most academically productive health centres in North America, TAHSN organizations work together to advance and sustain high-quality patient needs, education, knowledge transfer, and research innovation.^2^

### Defining cross-hospital collaboration

Surprisingly, there is a lack of clear understanding of what collaboration between healthcare organizations means; leaders can at times find it complicated and confusing to bring multiple organizations together and facilitate knowledge sharing to co-design solutions, specifically in conditions where health human resources remains a challenge.^3^ However, well-rehearsed literature suggests that cross-hospital collaboration improves patient outcomes, provides benefits to healthcare providers, reduces work load, and increases job satisfaction.^4^ The shared ownership of common goals, decision-making processes, and the integration of professional knowledge is linked to the following perceived benefits: (1) increased assessment quality, (2) reduced overall costs borne by all partners and the community at large through shared resources, and (3) strengthened trust and relationships among hospitals, its people, and communities.^5^

In practical theory, collaboration in healthcare is a dynamic, transforming process of creating a power-sharing partnership between professionals and institutions with diverse backgrounds and mandates who work together to provide high-quality healthcare while keeping a supportive and productive work environment.^3^ Definitions in the literature indicate that collaboration between hospitals can be described as the following:

• A coordinated system of synergistic alliance where all members work equitably together to implement a collective action plan; and through communication, negotiation, trust and respect between one another, are able to achieve common goals.^3^
• A merger between two or more organizations to develop common strategic purposes that promote organization’s mission and enhances organizational performance. The partners in the merger address common issues through mutual accountability and shared assets.^6^
• An open exchange of knowledge across multiple organizations, a culture of recognition of each other’s competence, and the sharing of information to accelerate operational mechanisms and successfully drive organizational change, transformation, and process improvement.^7^
• A process of working together between two or more healthcare professionals who have specific roles, perform interdependent tasks, and share a common vision, guided by a negotiated agreement which values expertise and contribution that each individual brings to deliver high-quality healthcare.^8^

## Methodology: Our collaborative approach

Gaps in collaboration between hospitals have been documented for decades and can result in a disjointed continuum of care as well as missed opportunities to address complex health issues.^9^ The COVID-19 pandemic was the most extreme public health crisis confronted by healthcare organizations worldwide. The unknown nature of the disease and the successive waves of the pandemic outstripped hospitals available resources and equipment, placing health systems under unprecedented pressure. Despite global efforts to limit the spread of the disease, hospitals continued to face challenges in executing appropriate responses which significantly impacted organizational readiness and success.

To address the strain in health human resources and inconsistencies in standards across OHS in response to the health emergency of international concern, the TAHSN organizations were encouraged to prioritize intentional collaboration to improve service quality and workforce safety. Using a case study approach, data was collected from the 14 healthcare organizations to study current state operational mechanisms in support of an enhanced, future state service delivery model; to launch programs and services shared between TAHSN organizations; establish consistent measures to cope with the pandemic; and consider actions for future preparedness to potential forthcoming crises. The ongoing, unified efforts toward standardization across the TAHSN organizations aimed to form an ecosystem that demonstrates excellence in strategic collaboration and impact to better solve health needs.

Measuring high in potential, reach, and impact, the collaborative expanded across the total TAHSN workforce of approximately 99,492 staff. While this figure represents a modest number of staff for all 14 organizations, the said data does not include volunteers, researchers, learners, and physicians who also rely heavily on OHS departments.

### Origin and initial proposal

In December 2021, the TAHSN Vice Presidents of Human Resources (VPHR) unfolded a fresh perspective, willingness, and commitment to connect OHS across the TAHSN healthcare organizations and generate consistency, quality, and innovation in best practice guidelines. This was an agile leadership approach to design fluid and dynamic interactivity between the organizations and foster standardization of processes and policies. To operationalize the shared goals, a network of OHS directors was established representing the 14 healthcare organizations: University Health Network; Sunnybrook Health Sciences Centre; Women’s College Hospital; North York General Hospital; The Hospital for Sick Children; Scarborough Health Network; Sinai Health System; Unity Health Toronto; Trillium Health Partners; Humber River Regional Hospital; Michael Garron Hospital; Baycrest Centre for Geriatric Care; Holland Bloorview Kids Rehabilitation Hospital; Centre for Addiction and Mental Health. Participation was contingent on a signed letter of commitment from TAHSN CEO and VPHR group. All organizations provided funding secured for an OHS project manager role that served as a central point person and supported with plan, design, and delivery of activities identified for the collaborative.

Over a 9-month period, a detailed review of existing OHS practices was completed to identify unique practices, strengths, and challenges and use the data to discover unforeseen opportunities for partnership. An overarching long-term improvement plan (from 2022 to 2025) was released that offered the foundation from which the TAHSN organizations can achieve the shared vision to (1) establish consistent policies and standards in an effort to enhance the OHS function across the continuum of care and (2) address areas of common concern that present the highest need for advanced solutions. In the first 2 months, systematic data was collected from OHS frontline staff to study current state operations within individual organizations; data collection addressed key elements that make up an optimal service delivery model: unique resources, services, systems, standards, infrastructure, and customer satisfaction. Workshops were held with the OHS director group over the course of 6 weeks to facilitate strategic planning, conduct prioritization mapping, and create a functional network of work streams to address persisting issues and achieve collective excellence in healthcare. Best practice recommendations were developed using both the findings from needs assessment (i.e., qualitative interviews) as well as key expert perspective, judgement, and continued experience of the OHS directors. As an example, if the findings from the assessment suggested that workload within OHS is labour intensive resulting in increased administrative burden and staff burnout, a recommendation was put forth to explore opportunities to automate workflows and identify system-level interventional strategies to promote health, well-being, and joy in work, respectively. Additionally, if there was a pressing need to standardize learning and development across the TAHSN organizations to promote and protect workforce well-being, the OHS leaders proposed establishing a centralized hub to foster education, training, and resources on topics such as mental health and workplace violence, facilitating early detection and prevention in a workforce and providing effective resources and tools to staff when confronted with such issues.

To implement best practice guidelines and deliver effective care based on existing knowledge and evidence, four broad strategic priorities were discovered, each integrating a subset of common goals. These priorities became the first step in the intentional journey of integrated effort to co-create processes and policies for a preferred future, through shared decision-making, resource allocation, and performance measurement:

1. **Establish a shared, future state service delivery model(s):** The TAHSN healthcare organizations will collaborate to ensure employees, learners, and volunteers receive the same level of service.
2. **Establish a collaborative framework across TAHSN:** The TAHSN healthcare organizations will promote consistency in practices through shared resources, expertise, and knowledge to reach an ideal state that will strengthen the defensibility of TAHSN programs.
3. **Establish best practices and standards to accelerate operational excellence holistically, including wellness and occupational safety:** The TAHSN healthcare organizations will create a mechanism to surface critical processes, evaluate these processes, and engage the right stakeholders to establish best practices.
4. **Develop a technology strategy and explore ways of enhancing workflows through automation to reduce administrative burden and staff burnout:** The TAHSN healthcare organizations will optimize relationships with internal and external stakeholders to develop a technology strategy that will explore opportunities to enable system functionalities and automate workflows.

### Standing up a multidisciplinary team structure

**Figure 1.**
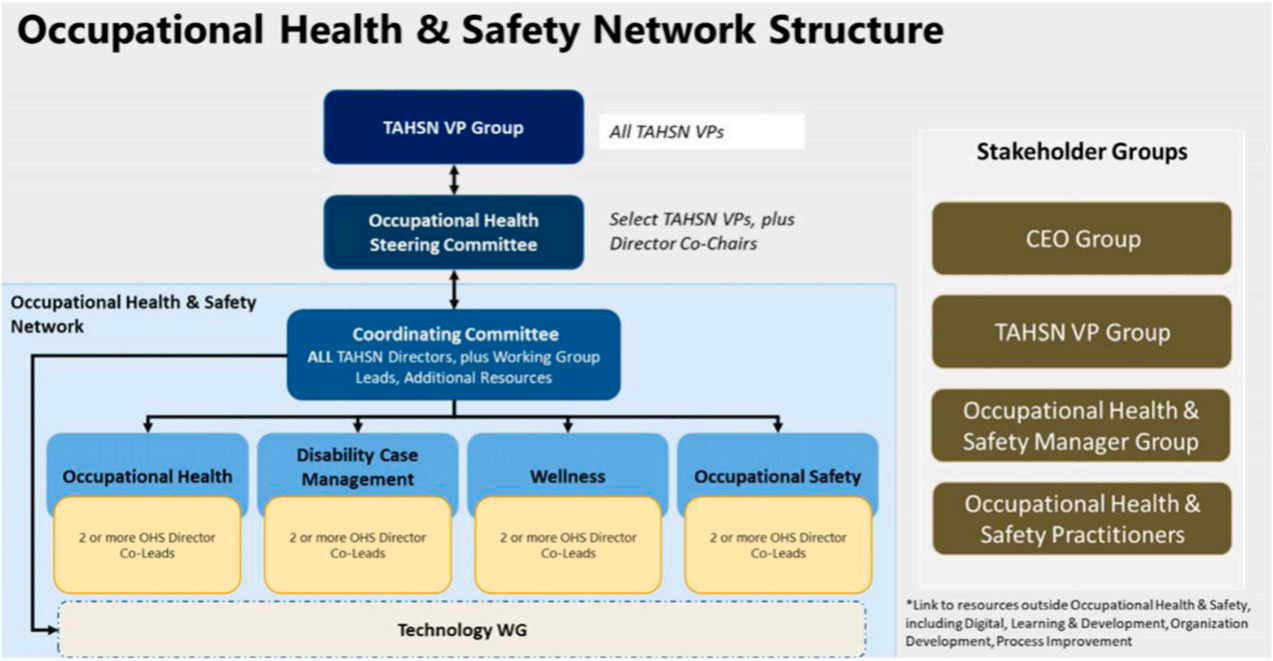
TAHSN collaborative network structure.

**Figure 2.**
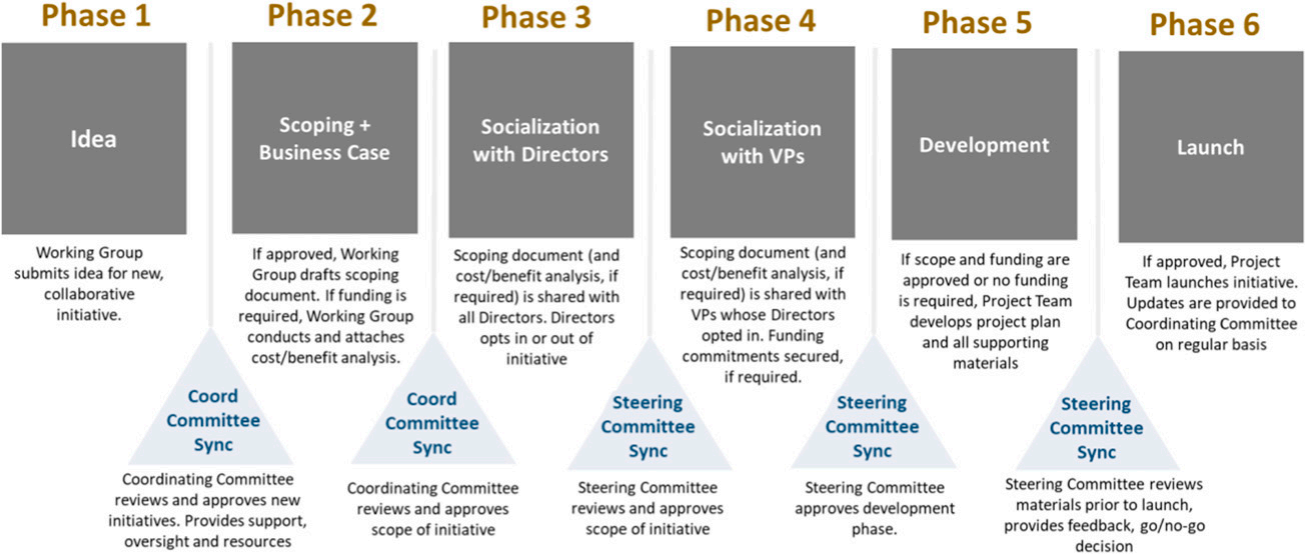
Collaborative pipeline.

A robust structure was designed to deliver the multi-year improvement plan that delineated a systematic, well-planned implementation process and support leaders in informed decision-making to enhance future state service delivery. Four work streams were stood up to achieve the strategic goals, objectives, and outcomes, each corresponding to the functional area within OHS: occupational health clinic, disability case management, occupational safety and wellness, guided by two or more OHS director co-leads who had the shared accountability to oversee day-to-day operational activities in alignment with the TAHSN collaborative network (See Figure 1) and collaboration pipeline (See Figure 2).

Membership of the four work streams consisted of 66 frontline staff members across the 14 healthcare organizations, making up a multidisciplinary team composition of subject matter experts and varying in roles including (but not limited) to managers, occupational health nurses, disability case managers, attending support consultants and return to work specialists. Selection of staff members was determined by the OHS directors and based on knowledge, experience. and skillset that would guide the design, review, and development of the team-based deliverables and benefits realization.

### An action-oriented roadmap to a preferred future

Upon the launch of the four work streams, an additional in-depth assessment was performed for each functional area influencing the domain in the present. The assessment was a culmination of extensive exploration and information synthesis of existing processes and structures from staff at the forefront of business operations who can offer an expanded focus to discover innovative tools, practices, and standards for ongoing strategic collaboration. The assessment was organized in a questionnaire format and developed to answer the questions across the following categories: (1) staffing model: what is the resource model/staffing ratio (e.g., how many occupational health nurses are staffed for clinic-related duties to serve the total volume of employees); (2) infrastructure: what facilities and hours of operations are available for established services and programs (e.g., fitness centres); (3) services: what services are provided to staff and are also generating revenues to improve financial performance (e.g., mask fit testing, gym membership); (4) technology: what technology is enabled to automate workflows and reduce administrative burden (i.e., booking models and new hires onboarding forms); and (5) practices: which practices/programs are most structured and receptive by staff and what are the challenges and gaps at the forefront of enhanced performance (e.g., peer support and mental health trainings).

This level of transparency enabled the TAHSN healthcare organizations to dive deeper, identify previously invisible performance gaps, and explore opportunities that result in evidence-based best practices. Based on the findings, the healthcare organizations capitalized on high performing services, programs, and practices currently in place and through a cost-sharing model, developed a roll out plan among organizations that committed an interest. For example, if one hospital had a quick turnaround on completing immunization status for new hires, the collaborative turned to that organization for guidance on systems and workflows that result in process efficiencies and more positive outcomes. If another organization had a particularly established wellness program (e.g., peer support and virtual fitness class calendar), the collaborative was invited to co-partner with the organization to achieve both cost efficiencies and standing up of shared programs that cultivate a culture of support and community building.

**Table:** 

Work stream	Shared goal
Occupational health clinic	Standardize occupational health clinic best practices and explore efficiencies within clinics to drive quality improvement
Disability case management	Develop a TAHSN disability management administrative manual that embeds recommended best practices of the absence management strategy
Occupational safety	Examine core services within occupational safety (i.e., workplace violence practices and training) through the lens of the strategic priorities
Wellness	Integrate, optimize, and align on solutions that enhance the health and well-being of staff across TAHSN healthcare organizations

## Results: Lessons learned and proposals for the future

Although healthcare organizations recognize the need to develop innovative partnerships to address pressing health needs, there is a sense of anticipation about the potential impact of collaborative efforts.^10^ Though establishing and maintaining any type of partnership model requires extensive commitment, we learned from our experience that a purposeful collaborative can be successful, and result in promising health outcomes when rooted in the following: a clear, shared vision that aligns partners towards a common goal; highly supportive leadership that promotes trust and confidence among team members by advocating for shared practices through mutual consensus; and a rigorous pursuit of innovative change that demonstrates perseverance during challenging times. Literature suggests that lack of collaboration in healthcare settings contributes to sub-optimal care.^11^ However, cross-hospital collaboration is an effective approach for sharing information and creating common goals which is considered fundamental not only for delivering high-quality healthcare but also a community-wide impact and informed decision-making among leaders. As the TAHSN healthcare organizations progressed through the collaborative, there were valuable lessons identified that have the potential to assist other organizations as they develop similar, shared initiatives.

**Figure 3.**
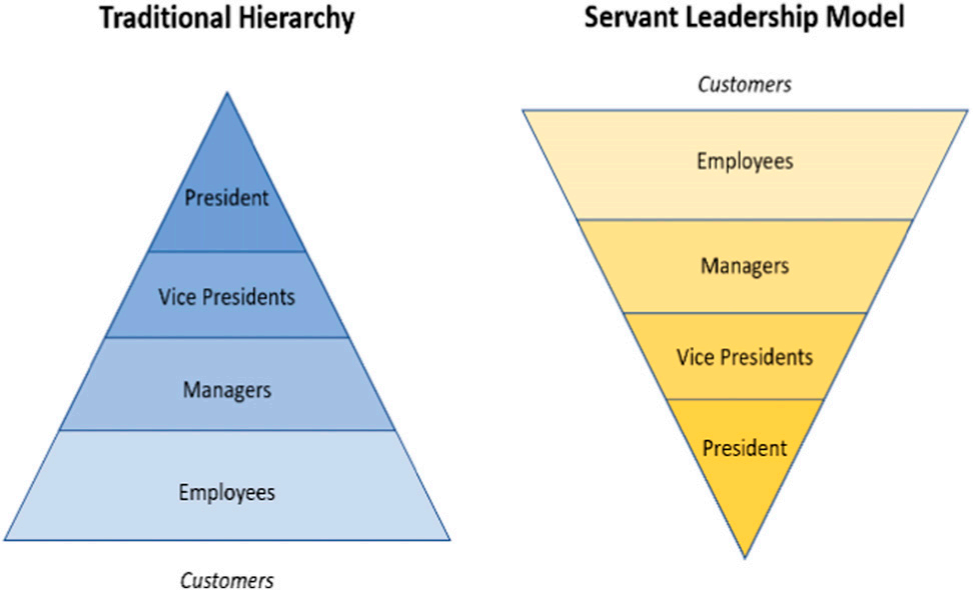
Servant leadership model.

1. Competition between healthcare organizations can exacerbate inequities in service delivery. On the contrary, partnership fosters an innovative merger where leaders can combine capabilities to improve access to and delivery of state-of-the-art healthcare; and through collective strength and excellence, delineate, co-design, and implement practices and management strategies that enable health systems to better respond in times of crisis.
2. Collaboration carries a positive resonance and allows organizations to carry an abundance mindset, as opposed to a scarcity mindset, challenging them to view partnership as a “win-win” opportunity. When organizations work together, they are able to better discover performance of business targets and understand where individual organizations stand relative to their peers. Through performance mapping, health leaders can identify high performing services and capitalize on existing programs that are working well at a local level to develop evidence-informed best practices.
3. Bending the curve of health status in a community requires significant amount of effort and dedication, specifically through shared authority and responsibility. Trust, respect and teamwork are central to a system of working together to achieve common goals. Constituting a collaborative practice between organizations specifically requires the following (a) nurturing an ecosystem that focuses on coordination and transparency to create a shared vision and (b) clear communication, negotiation, and cooperation between all partners to showcase achievement and strengthen community building.
4. Cross-hospital collaboration adopts the servant leadership model, introduced by Robert K. Greenleaf and is an important attribute of a collaborative. Rooted in shared power, consensus building, and influence, servant leadership is a form of moral-based leadership style, where the goal of the leader is to prioritize needs of the workforce and ensure growth and well-being of people and communities to which they belong to; the desired outcome is then an empowered workforce and an organizational culture of support and recognition.^12^ This is different from a traditional leadership style that involves accumulation and exercise of power by one at the “top of the pyramid” (see Figure 3); the focus of a traditional leader instead is the thriving of the organization.
5. Integrating a cost-sharing model into program design strengthens stakeholder commitment, participation, and readiness for process improvement. When organizations in a partnership share cost of interventions, they are more likely to feel a sense of ownership and responsibility which empowers them to drive ongoing growth beyond the implementation phase. This self-sustaining approach fundamentally contributes to long-term success, measuring high in impact and sustainability of shared programs and services. The cost-sharing model is also beneficial in a partnership as it reduces the burden of operational cost for a program on a single party and ensures that sharing of costs, benefits, and responsibilities are equitably distributed among all stakeholders.
6. Health initiatives require considerable time to achieve improvements, specifically if the initiative is dependent on organizational transformation, change or process efficiencies. In a collaborative, where members share their unique perspectives and knowledge to develop novel solutions together, celebrating short-term successes and rewarding employees for their contributions cultivates a workplace culture of support and increases organic growth and productivity, leading to improved engagement and higher retention.
7. Developing a collaborative pipeline that serves as a guiding document of mutual consensus is an effective and clear process of collective decision-making and leadership oversight. This process enables a framework of more deliberate, thoughtful decisions, advocating for building synergistic alliances that are rooted in trust, equity, and inclusivity. Fundamentally, the framework ensures that staff generated initiatives are well-designed, well-resourced, and have the highest likelihood of success possible.
8. Project management is a dynamic discipline that can considerably enhance the efficiency and effectiveness of a collaborative, positively impacting its nature, culture, and success. Integrating this process helps teams remain organized and on track to successfully accomplish goals, objectives, and outcomes. Other advantages of project management to support ongoing strategic collaboration include improved communication and change strategies; more positive stakeholder relationships; a structured process for risk escalation and management; and an overall more productive environment for teamwork.

## Discussion

The OHS collaborative across the 14 TAHSN healthcare organizations was the first step to a meaningful journey of integrated effort to lead the future of healthcare and develop innovative solutions that will increase strength and defensibility of TAHSN programs. Establishing a multidisciplinary team of 66 subject matter experts allowed for trust, respect, and confidence to develop between organizations and successfully drive uniformity and standardization of practices and shared programs. Secondly, by establishing a clear and shared vision for OHS, the healthcare organizations released an overarching improvement plan that delineated forward thinking, objective setting, prioritization mapping, and strategic collaboration to implement both short and long-term solutions that will enhance the existing service delivery model in the Toronto region. The improvement plan became the cornerstone for this purposeful collaborative to showcase effort and planned achievements through collective strength and excellence. Thirdly, the launch of the four work streams representing the OHS functional domains allowed the TAHSN organizations to work together, identify unique strengths and challenges, and capitalize on “high performing” services through shared cost and resources (i.e., toolkits, e-learning modules, and virtual fitness classes). Standing up a structured, governing network of OHS directors provided leaders with a platform to remain connected through a shared digital hub, continue to identify opportunities to partner on, and combine efforts to efficiently standardize processes (e.g., explore opportunity to align on communicable disease surveillance programs; medical and human rights accommodation (i.e., PAPR) for post-graduate learners; occupational safety trainings; and mental health programming).

An environment scan was conducted during the current state needs assessment to explore and study the operational mechanisms, including staffing models of OHS at a national level. Although one study highlighted that the OHS departments in British Columbia are found to be under-resourced and under-staffed, there were no established or staffing guidelines proposed in the literature to address the constraints on health human resources.^13^ Qualitative research conducted across the 14 TAHSN healthcare organizations suggested the persisting need to adequately resource OHS departments in proportion to the volume of programs and services offered to reduce or buffer the negative effects of increased work that causes physical and emotional exhaustion, leading to work stress and staff burnout.

The pandemic has brought to the forefront the importance of a consistent service delivery across the TAHSN healthcare organizations that not only offers highest quality of healthcare but also contributes to both physical and psychological well-being of the hospital workforce. This intentional collaborative unfolded the novel thinking of establishing common goals for ongoing strategic partnership and co-designing practices that accelerate operational performance and address system-level constraints to support HCWs. By combining knowledge, experience and perspectives, the TAHSN organizations have embarked a rewarding journey to rigorously pursue implementation of shared interventional strategies and programs to enable transformation, change, and process improvement that fosters an organizational culture of support and recognition. TAHSN organizations are determined to continue strategic collaboration to develop initiatives that keep workers healthy and safe.


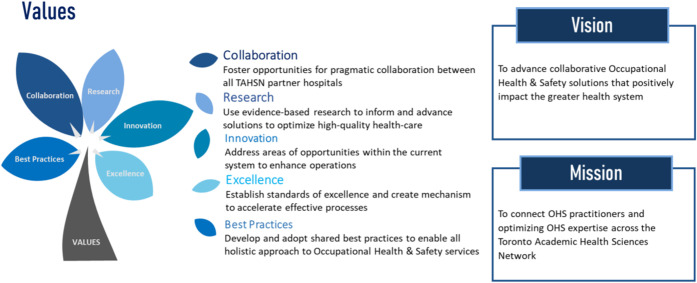


## Conclusion: Our final word

Collaborative community health efforts are cited as beneficial to those who participate. Although achieving a synergistic relationship relies on a number of factors, the benefit to the community by combining knowledge, resources, and skills is greater than what can be achieved alone.^9^ Through collaborative efforts, there is a greater potential for improved health outcomes at a broader level.^9^

Hospitals are encouraged to step out of their comfort zones and participate in collaborative initiatives as a way to demolish and flatten a “silo” approach. To foster a collaborative that is rooted in collective strength, excellence, research, and discovery, healthcare organizations are recommended to have a congruent perspective on how and why collaboration is of value. To break down barriers of joint ventures and implemented integrated efforts, it is important to identify and measure a common language of collaboration by establishing a clear, shared vision.^9^ When a shared vision and common goals exist, resources are better able to work together and find optimal ways of realizing benefits in the most time and cost effective manner. Process improvement initiatives that require identifying community-wide health needs, determining priorities, and developing long-term improvement plans to collectively develop advanced solutions are specific approaches that begin to break down the silo approach that stand in the way of innovation and organizational development.

The reluctance for cross-hospital collaboration may be due to the uncertainty and apprehensiveness leaders feel to place resources in an already constraint environment when it comes health human resources and budget allocation. However, abundance mindset allows leaders to adopt innovative management strategies while recognizing that shared resources and cost can launch services that have the ability to create the highest impact at a community level and also improve financial performance of individual organizations. Although resources are scarce within hospitals, quantifying the specific levels of action necessary based on priority areas may lead to better determining an action-oriented roadmap for a preferred future. Communicating common goals, creating shared accountabilities and building alliances together are effective strategies of shifting mindsets and encouraging resources to work together.^13–15^

### Limitations

Managing a large group of stakeholders in leadership positions and across multiple organizations means planning for perceived resistance while aligning on common goals and constraints around stakeholder availability and bandwidth. Developing a communication plan in early stages that provides balanced and objective information on goals, outcomes, and responsibilities is necessary to set expectations and avoid anticipated schedule delays. A dedicated project management resource that serves as a central point person between all stakeholders is important for achieving intended outcomes.
